# Editorial: Crucial Decisions in Severe Traumatic Brain Injury Management: Criteria for Treatment Escalation

**DOI:** 10.3389/fneur.2021.740915

**Published:** 2021-09-21

**Authors:** Chiara Robba, Mathieu van der Jagt

**Affiliations:** ^1^Anesthesia and Intensive Care, Policlinico San Martino, IRCCS for Oncology and Neuroscience, Genova, Italy; ^2^Dipartimento di Scienze Chirurgiche ed Integrate, University of Genova, Genova, Italy; ^3^Department of Intensive Care Adults, Erasmus MC – University Medical Center, Rotterdam, Netherlands

**Keywords:** intracranial hypertension, traumatic brain injury, brain injury, treatment, staircase

Intracranial hypertension [IH, or too high intercranial pressure (ICP)] is a major cause of secondary brain damage and is associated with poor outcomes in traumatic-brain-injured patients (1–3). For this reason, IH should be promptly and aggressively managed to optimize chances for recovery. Therefore, monitoring and treatment of ICP and cerebral perfusion pressure (CPP) has become the cornerstone of severe TBI management (1, 2).

Several basic and more aggressive interventions are available to treat IH (4, 5). However, increasing level of intensity treatment is associated with higher incidence of adverse events and risks (4, 5). In general, IH management follows a staircase approach, with the higher intensity of treatment being associated with the highest risks.

A recent Delphi-method-based consensus (6) aimed to establish an updated TBI protocol for the management of ICP in adult patients. A panel of 42 experts defined different ICP management protocols, consisting of three tiers of therapies with escalating treatments and risks in order to help clinicians in the management of patients with increased ICP.

Factors that may aggravate IH include not only intracerebral, but also extracerebral issues such as respiratory problems, hypotension, hyponatremia, seizures, obstruction of venous outflow, and fever (5).

Therefore, the maintenance of physiological homeostasis represents the first-line therapeutical tool, and this includes a number of basic measures such as elevation of the head to 30 degrees, hemodynamic stability with maintenance of euvolemia, appropriate comfort and sedation, stabilization of the airways, and mechanical ventilation to maintain appropriate targets of oxygen and carbon dioxide.

Hyperpyrexia should be avoided, and antiepileptic drugs should not be administered as prophylactic measures.

If basic measures are not sufficient to control ICP, a higher level of intensity may be considered (5).

Osmotic agents (such as mannitol and hypertonic saline), for instance, can reduce intracranial pressure and reduce brain volume by creating a gradient across the blood–brain barrier, but they can have several side effects, such as polyuria, alterations of plasma osmolarity, and electrolytes disturbances (4, 5).

In the case of refractory IH, more aggressive second tier therapies can be used, although these present important side effects.

Hypocapnia consequent to hyperventilation is a potent modulator of pial arterioles, causing cerebral vasoconstriction and therefore reduction of intracranial volume and ICP (7). However, because of the risk of cerebral ischemia, hyperventilation is currently indicated for the reduction of elevated ICP only in patients at risk of imminent cerebral herniation (8).

Therapeutic hypothermia can reduce cerebral metabolism and cerebral blood volume (4). Mild hypothermia (32–34°C) may effectively decrease intracranial pressure (9). However, recent evidence demonstrated that early or prophylactic hypothermia can be detrimental in TBI patients (9, 10).

As another example, decompressive craniectomy, although it can rapidly reduce ICP in cases of refractory IH, can improve mortality, but can also lead to a higher rate of unfavorable neurologic outcome (11).

The increasing risk for treatment-related complications of ICP-reducing therapies increases from basic to higher level of intensity treatment. It is therefore fundamental that the clinicians consider the risks and benefits associated with each strategy for escalation of treatment, and consider patient- specific pathophysiological features (12–15).

In this special issue, we collected articles that focus on the treatment escalation decisions in severe TBI.

Biomarkers and imaging are important in TBI management, as exemplified by the articles by Pinggera et al., Cardim et al., and Lenstra et al. These studies show the feasibility and safety of early MRI, which is informative for clinical practice, assess differences between optic nerve sheath diameter in healthy volunteers compared with TBI patients, and show that ECG abnormalities associated with TBI may represent prognostic biomarkers rather than intrinsic cardiac disease.

Rasulo et al. studied the association of lactate-pyruvate ratio in perilesional areas in intracerebral hemorrhage and cerebral autoregulation in an international multicenter study, showing the interdependence of cerebral metabolism and hemodynamics. This cerebral-systemic interdependence is also shown by Robba et al. who found that pulmonary pathologies impacting on oxygenation impacts on prognosis in TBI patients.

Further, several reviews and an opinion article assess the current insight into treatment escalations in TBI. Lazaridis provides new insights in how to leverage treatment utility and patient preferences in the difficult decision making regarding decompressive craniectomy as a rescue therapy for refractory IH. Finally, Battaglini et al., Gouvea Bogossian et al., and Godoy et al. review treatment escalations en de-escalations for TBI management and recalibrate the current status of hyperventilation as a management option.

The manuscripts included in this collection aim to add important clinical insights and help physicians in decision making for ICP management and escalation of treatment. In our opinion, the collected articles can add important knowledge to current neurocritical care literature and help with “crucial decisions” for TBI management.

## Author Contributions

All authors served as guest editors for this article collection and contributed equally to this Editorial.

## Conflict of Interest

The authors declare that the research was conducted in the absence of any commercial or financial relationships that could be construed as a potential conflict of interest.

## Publisher's Note

All claims expressed in this article are solely those of the authors and do not necessarily represent those of their affiliated organizations, or those of the publisher, the editors and the reviewers. Any product that may be evaluated in this article, or claim that may be made by its manufacturer, is not guaranteed or endorsed by the publisher.
